# Cognitive, emotional, and quality of life outcomes in patients with pulmonary arterial hypertension

**DOI:** 10.1186/1465-9921-7-55

**Published:** 2006-03-31

**Authors:** Joanne White, Ramona O Hopkins, Eric W Glissmeyer, Natalie Kitterman, C Gregory Elliott

**Affiliations:** 1 Psychology Department, Brigham Young University, Provo, Utah; 2 Neuroscience Center, Brigham Young University, Provo, Utah; 3 Department of Medicine, Pulmonary and Critical Care Divisions, LDS Hospital and the University of Utah, Salt Lake City, Utah

## Abstract

**Background:**

The effects of pulmonary arterial hypertension on cardiovascular and physical function are well documented. Limited information exists regarding the effects of pulmonary arterial hypertension on cognitive function despite patient reports of problems with memory and attention. Our primary purpose was to determine if a prospectively identified cohort of pulmonary arterial hypertension patients had cognitive sequelae. Our secondary purpose was to determine the relationships between cognitive sequelae and neuropsychological test scores with depression, anxiety, and quality of life.

**Methods:**

Forty-six adults with pulmonary arterial hypertension underwent assessment of cognitive function, depression, anxiety, and quality of life using standardized neuropsychological tests and questionnaires. The patients' scores were compared to normal population data. Medical, affective, neuropsychological, and quality of life data for patients with and without cognitive sequelae were compared using analysis of variance, Chi-square, or Fisher exact tests for categorical data. Correlations assessed relationships between neuropsychological test scores, depression, anxiety, quality of life, and medical data.

**Results:**

Cognitive sequelae occurred in 58% (27/46) of the pulmonary arterial hypertension patients. Patients with cognitive sequelae had worse verbal learning, delayed verbal memory, executive function, and fine motor scores compared to patients without cognitive sequelae. Twenty-six percent of patients had moderate to severe depression and 19% had moderate to severe anxiety. Depression, anxiety and quality of life were not different for patients with or without cognitive sequelae. Our patients had decreased quality of life, which was associated with worse working memory.

**Conclusion:**

Patients with pulmonary arterial hypertension have cognitive impairments, depression, anxiety, and decreased quality of life. Depression, anxiety, and quality of life were similar for patients with cognitive sequelae compared to those without cognitive sequelae. Decreased quality of life was associated with worse verbal and working memory. Clinicians should be aware of adverse brain related outcomes in PAH patients. Attention to proximal determinants and possible interventions to prevent or reduce cognitive and emotional morbidity and decreased quality of life are warranted and should be an emphasis in outcomes research.

## Background

Pulmonary arterial hypertension (PAH) are a group of diseases where obstructive pathologic changes occur in the small musculature of the pulmonary arteries [1]. Pulmonary arterial hypertension is characterized by shortness of breath, chest pain, edema, and syncope and is associated with life-threatening right heart failure [2]. Diagnostic classification includes familial PAH and idiopathic PAH which occurs in association with collagen vascular disease, congenital systemic to pulmonary shunts, portal hypertension or exposure to drugs and toxins. Although the pathogenesis of PAH is not completely understood, widely accepted mechanisms include vasoconstriction, endothelial dysfunction and smooth muscle cell proliferation, and thombosis [3]. Therapies such as calcium channel antagonists, prostacyclin analogues, phosphodiesterase-5 inhibitors, and endothelin receptor antagonists have reduced mortality, improved functional abilities, and improved quality of life [4].

Studies of the natural history of PAH found the median length of survival was 2.8 years [5]. While PAH patients are living longer, decreased exercise tolerance [6], reduced energy, and limited physical mobility are common [7]. Depression, anxiety, and decreased quality of life are also reported [7]. While the effects of PAH on cardiovascular and physical function are well documented, less is known regarding the effects of PAH on the brain and cognitive function despite patient reports of problems with memory and attention [8]. Cognitive function, emotional function, and quality of life are important as many PAH patients are living longer and may develop brain related morbidity.

We previously reported that telephone administered neuropsychological tests to assess cognitive function in PAH patients were reliable and valid [9]. Cognitive impairments and their associations with depression, anxiety, and quality of life in patients with PAH have not been described. We reasoned that PAH patients may have similar cognitive impairments to those observed in disorders characterized hypoxemia [10-12] and right heart failure [13]. Our primary purpose was to determine if a prospectively identified cohort of patients with PAH have cognitive sequelae. Our secondary purpose was to determine the relationships between cognitive sequelae and neuropsychological test scores with depression, anxiety, and quality of life.

## Methods

### Study subjects

Consecutive PAH patients were prospectively recruited from the pulmonary hypertension clinic at LDS Hospital, in Salt Lake City, Utah. LDS Hospital is a major referral and tertiary care center with a moderately large PAH population. Eligible PAH patients diagnosed by PAH by a board certified pulmonologist (CGE) based on clinical and laboratory data, and the 2003 World Health Organization classification guidelines were evaluated for this study. Study exclusion criteria were prior traumatic brain injury, neurologic disease (i.e., multiple sclerosis, stroke, dementia, etc.), psychiatric disorders with psychosis, co-morbid disease with known cognitive impairments (i.e., HIV, chronic obstructive pulmonary disease, coronary artery bypass surgery, cerebral ischemia), non-English speaking, and pulmonary hypertension not PAH. The LDS Hospital Institutional Review Board approved this study and it conformed to institutional and federal guidelines for the protection of human subjects. All patients signed statements of informed consent.

### Study design and methods

There were 95 patients with pulmonary hypertension of which 30 were excluded, due to diagnosis not PAH (n = 28) and English not their primary language (n = 2). Of the 65 eligible PAH patients 15 declined the study, 2 had medical complications, 2 we were not able to contact, and 2 patients died prior to study enrolment resulting in 46 enrolled patients. All patients received normal clinical care and treatment in the pulmonary hypertension clinic. Patient medical data were collected prospectively as part of routine clinical care. The medical data included New York Heart Association (NYHA) functional classification, medications, supplemental oxygen use, 6-minute walk data, Borg dyspnea index, and laboratory data. Demographic data including age, gender, education level, and psychiatric history were obtained as part of the neuropsychological assessment. In order to ensure the patient's oxygen levels were within the normal range during neuropsychological testing, pre- and post-testing oxygen saturation (SpO_2_) and heart rate were obtained using a Nonin pulse oximeter [14]. Patients that required oxygen therapy were tested on supplemental oxygen.

### Cognitive outcome

Standardized neuropsychological tests were administered in a quiet private office at LDS Hospital. The neuropsychological tests assessed general intelligence, attention, memory, mental processing speed, executive function (i.e. decision making, organization, problem solving, etc.), and fine motor abilities. The neuropsychological tests are sensitive to cognitive impairments, with known reliability and validity and are widely used in a variety of clinical, medical, psychiatric, and research populations.

General intellectual functioning was assessed using the Weschler Abbreviated Scale of Intelligence (WASI; mean = 100, SD = 15) [15]. To determine the possible effects of PAH on general intellectual functioning, the National American Adult Reading Test (NAART) was used to assess premorbid intellectual function [16]. The NAART is a quickly administered index that is widely used to estimate intellectual ability. The predicted intellectual function from the NAART can be compared with current performance to determine the extent of intellectual decline. The NAART has been shown to be valid in estimating premorbid intellectual function in a variety of neurologic populations [16]. The NAART yields intelligence quotient (IQ) score (mean = 100, SD = 15) and the estimated premorbid IQ score was compared to the measured Full Scale Intelligence Quotient (IQ) from the WASI.

Memory was assessed using the Logical Memory I and II subtests from the Weschler Memory Scale-III (WMS-III) [17], Rey Complex Figure Test [18], and Rey Auditory Verbal Learning Test [16]. Executive Functioning was assessed using the Trail Making Test Parts A and B [19], Wisconsin Card Sorting Test [16], and Hayling Sentence Completion Test [20]. Attention was assessed using the Stroop Test [16], Ruff 2&7 Selective Attention Test [21], and Digit Span from the WMS-III. Mental processing Speed was assessed using Symbol Digit subtest from the WMS-III. Motor function was assessed using the Grooved Pegboard [22], and Finger Tapping tests [22]. Scores were calculated using published population normative data for each test and were transformed to age and gender corrected T-Scores [16,23]. T-scores have a mean of 50 and standard deviation of 10 and allow for comparison across cognitive test scores and patients.

Cognitive sequelae (presence or absence) was defined *a priori *as 2 or more neuropsychological test scores that were >1.5 SD or 1 test score that was >2 SD below the normative population data. Our definition of cognitive sequelae is similar to those used by others and ourselves in previous studies [24,25]. We also report the percent of patients with cognitive impairments for each cognitive domain (e.g. memory, mental processing speed, attention, executive function, and motor function).

### Depression and anxiety

Depression and anxiety were measured using the Beck Depression Inventory (BDI) [26] and Beck Anxiety Inventory (BAI) [27]. For the BDI scores of 0 to 9 indicate minimal depression, 10 to 16 mild, 17 to 29 moderate, and 30 to 63 severe depression. For the BAI scores of 0 to 7 indicate minimal anxiety, 8 to 15 mild, 16 to 25 moderate, and 26 to 63 severe anxiety.

### Quality of life

The Medical Outcome Study 36-Item Short Form Health Survey (SF-36) [28] was administered to assess quality of life. The eight domains of the SF-36 (physical functioning, role-physical, bodily pain, general health, vitality, social functioning, role-emotional, and mental health) are clustered to form two higher order domains, the physical and mental health scores. Each domain is scored from 0 to 100, with higher scores indicating better quality of life. The SF-36 has been used in a variety of patient populations and norms for age and gender are available [28,29].

### Statistical analyses

Descriptive statistics were carried out for demographic, medical, neuropsychological, and quality of life scores and are reported as mean ± standard deviation. Pre- and post-neuropsychological test oxymetric oxygen saturation (SpO_2_) and heart rate were compared using paired t-tests. The patient's measured Full Scale IQ (WASI) was compared to the estimated premorbid Full Scale IQ (NAART score) using paired t-tests. Cognitive sequelae are reported as percentage of patients. Data for depression (BDI) and anxiety (BAI) are reported as the percentage of patients.

Medical, affective, neuropsychological, and quality of life data for patients with cognitive sequelae and without cognitive sequelae were compared using analysis of variance (ANOVA) for continuous data and Chi square or Fisher exact tests for categorical data.

Pearson's Product Moment correlations were used to assess the relationships between neuropsychological test scores, depression, anxiety, and quality of life and medical data. Correlation coefficients were considered significant at a 0.01 two-sided significance level in order to reduce the number of spuriously significant findings resulting from multiple comparisons.

## Results

### Descriptive statistics

Descriptive statistics and medical data for the PAH patients are shown in Table 1. There were 46 PAH patients enrolled, 38 (82.6%) female, mean age of 48.2 ± 11.8 years (range 20 to 69 years) and a mean education level of 13.6 ± 3 years (range 4 to 18 years). The time from PAH diagnosis to neuropsychological testing was 2.6 ± 2.6 years. There were no significant differences between the PAH patients' pre-test SpO_2 _(92.7 ± 3.9%) and post-test SpO_2 _(92.5 ± 3.3%; p = 0.43) and pre-test heart rate (84.9 ± 13.2) and post-test heart rate (83.5 ± 14.4; p = 0.66).

**Table 1:** Demographic and Medical Data

	**Total Group** ** (N = 46)**	**Cognitive Sequelae** ** (N = 27)**	**No Cognitive Sequelae** ** (N = 19)**	**Cognitive Sequelae vs. No Cognitive** ** Sequelae P value**
Gender Female N (%)	38 (82.6%)	23 (60.5%)	14 (39.5%)	0.43
Age in years M ± SD (range)	48.2 ± 11.8 (20 to 69)	48.6 ± 11.8 (24 to 69)	47.6 ± 12 (20 to 65)	0.78
Education in years M ± SD (range)	13.6 ± 3.0 (4 to 8)	13.3 ± 3.3 (4 to 18)	14.1 ± 2.4 (11 to18)	0.37
Time from PAH Diagnosis to testing in years M ± SD (range)	2.6 ± 2.6 (0.08 to 11.9)	2.0 ± 1.4 (0.08 to 5.2)	3.4 ± 3.5 (0.08–11.9)	0.07
New York Heart Classification: N (%)				
Class I	2 (4.3)	2	0	0.17
Class II	9 (19.6)	5	4	0.47
Class III	34 (67.4)	11	23	0.10
Class IV	1 (2.2)	0	1	0.99
Etiology of PAH: N (%)				
Drug/Toxin	16 (34.8)	6	10	0.77
Primary Pulmonary Hypertension	19 (40)	10	9	0.23
Collagen Vascular Disease	8 (17.4)	3	5	0.82
Portal Hypertension	2 (4.3)	0	2	0.50
Congenital systemic-to- pulmonary shunt	1 (2.2)	0	1	0.50
Medications: N (%)				
Vasodilators	25 (54.3)	9	16	0.32
Calcium Channel Blockers	16 (34.8)	9	7	0.13
Endothelial Receptor Antagonists	15 (32.6)	6	9	0.59
Anticoagulants	30 (65.2)	13	17	0.48
Prostacyclins				
Epoprostenol	12 (25.5)	6	6	0.48
Treprostinil	16 (34.8)	10	6	0.71
Supplemental Oxygen: N (%)	30 (65.2)	10	20	0.11
Oxygen Daytime: liters/minute	2.84	3.0	2.6	0.54
Oxygen Night: liters/minute	2.90	2.9	2.8	0.84
Medical Variables M ± SD				
PaO_2_				
Diagnostic	63.7 ± 12.5	62.2 ± 15	65.6 ± 8.0	0.39
Most Recent (N = 30)	68.0 ± 9.8	66.3 ± 10	70.4 ± 9.3	0.27
Mean Right Atrial Pressure (mmHg)				
Diagnostic	12.1 ± 6.9	11.4 ± 5.7	13.0 ± 8.5	0.45
Most Recent (N = 34)	11.0 ± 5.4	10.9 ± 5.6	11.1 ± 5.1	0.92
Mean Pulmonary Capillary Wedge Pressure (mmHg)				
Diagnostic	11.4 ± 4.5	11.6 ± 4.8	11.1 ± 4.1	0.75
Most Recent (N = 33)	11.2 ± 5.3	11.4 ± 5.5	10.9 ± 5	0.79
Mean Pulmonary Artery Pressure (mmHg)				
Diagnostic	53.6 ± 13.9	52.1 ± 13.1	55.5 ± 15.2	0.42
Most Recent (N = 34)	52.7 ± 13.5	55.6 ± 13.7	48.8 ± 12.5	0.15
Mean Cardiac Output (thermo; L/min)				
Diagnostic	4.4 ± 1.8	4.2 ± 1.8	4.6 ± 1.8	0.48
Most Recent (N = 31)	4.5 ± 1.6	4.4 ± 1.5	4.5 ± 1.8	0.87
Mean Cardiac Index (thermo; L/min)				
Diagnostic	2.4 ± 0.8	2.2 ± 0.7	2.6 ± 0.7	0.27
Most Recent (N = 10)	2.4 ± 0.5	2.7 ± 0.4	2.2 ± 0.3	0.06
Pulmonary Vascular Resistance (Woods unit)				
Diagnostic	11.4 ± 5.2	11.6 ± 5	11.1 ± 5.4	0.77
Most Recent (N = 30)	11.1 ± 6.1	11.6 ± 6.6	10.4 ± 5.6	0.58

PAH = Pulmonary Arterial Hypertension

Diagnostic = Taken from data obtained at the time of PAH diagnosis

Most Recent = Taken from the patients most recent laboratory and right hearth catheterization data.

The PAH patient's estimated mean premorbid IQ (111 ± 9.1) was significantly higher than their measured mean Full Scale IQ (103 ± 12; t = 6.41, p < 0.0001). Fifty-eight percent (27/46) of the PAH patients had cognitive sequelae 2.6 years (mean) following diagnosis. In individual cognitive domains, 56.5% (26/46) had impaired motor function, 41.3% (19/46) had impaired memory, 17.4% (8/46) had slow mental processing speed, 15.2% (7/46) had executive dysfunction, and 13.5% (6/46) had impaired attention. Table 2 outlines neuropsychological test scores.

**Table 2:** Neuropsychological Test Scores

	**Total Group** ** M ± SD**	**Cognitive Sequelae** ** M ± SD**	**No Cognitive** ** Sequelae M ± SD**	**Cog. Sequelae vs. No Cog.** ** Sequelae P value**
WASI				
Full Scale IQ	103 ± 12.0	100 ± 13.5	107 ± 7.8	0.06
NAART Estimated Premorbid IQ				
Full Scale Intelligence Quotient	111 ± 9.1	109 ± 9.9	114 ± 6.9	0.13
**Memory**				
Rey Auditory Verbal Learning Test (Raw Scores)				
Trial 1	6.2 ± 1.9	5.7 ± 1.8	6.8 ± 1.7	**0.04**
Trial 5	12.3 ± 2.3	11.5 ± 2.5	13.3 ± 1.4	**0.01**
Immediate Recall	10.5 ± 3.2	9.7 ± 3.3	11.5 ± 2.8	0.07
Delayed Recall	10.3 ± 3.3	9.8 ± 3.6	10.8 ± 2.7	0.29
Logical Memory (Percentile)				
Immediate Recall	41.4 ± 27.8	32.5 ± 26.1	54.0 ± 25.6	0.08
Delayed Recall	41.9 ± 27.4	32.2 ± 24.4	55.8 ± 25.8	**0.03**
Rey Complex Figure Test (T-scores)				
Immediate Recall	49.8 ± 14.9	46.8 ± 15.1	54.1 ± 13.8	0.88
Delayed Recall	47.9 ± 14.7	45.4 ± 16.0	51.4 ± 12.0	0.13
**Attention**				
Letter-Number Sequencing (Scaled Score)	9.5 ± 2.8	8.9 ± 2.9	10.2 ± 2.4	0.27
Digit Span (T-score)	59.9 ± 10.4	58.6 ± 10.8	61.7 ± 9.7	0.32
Ruff 2&7 Test (T-scores)				
Total Speed	58.0 ± 10.9	56 ± 11.6	61.0 ± 9.3	0.11
Total Accuracy	44.8 ± 12.5	45 ± 14.7	44.5 ± 8.6	0.89
Stroop Color-Word T-score	48.2 ± 14.5	47 ± 14.5	49.8 ± 14.7	0.91
**Executive Function**				
Trail Making Test (T-scores)				
Part A	50.3 ± 10.1	49.3 ± 10.3	51.6 ± 9.8	0.45
Part B	49.5 ± 7.9	49.5 ± 7.7	49.5 ± 8.3	0.98
WCST				
Total Score (T-score)	46.1 ± 9.9	44.8 ± 8.2	48.1 ± 12	0.28
Perseverative Errors (T-score)	48.4 ± 7.9	46.4 ± 8.4	51.4 ± 6.0	**0.04**
Number Categories Completed	5.2 ± 1.3	4.9 ± 1.4	5.6 ± 0.8	0.07
Hayling Sentence Completion Test				
Total Score (Scaled Score)	5.6 ± 1.7	5.5 ± 2.0	5.8 ± 0.9	0.55
**Mental Processing Speed**				
Symbol Digit (Raw Score)	52.6 ± 13.8	51.7 ± 14.8	50.8 ± 17.5	0.86
**Motor Function **(T-scores)				
Finger Tapping Right hand	58.1 ± 12.1	55.4 ± 12.9	62.6 ± 9.4	**0.05**
Finger Tapping Left hand	61.7 ± 9.3	59.4 ± 8.2	65.9 ± 9.9	**0.03**
Grooved Peg Board Right hand	43.7 ± 11.5	37.8 ± 10.4	53.2 ± 5.0	**<0.001**
Grooved Peg Board Left hand	42.8 ± 12.7	41.0 ± 13.8	46.1 ± 9.7	0.21

Neuropsychological scores are presented as Mean ± Standard Deviation (M ± SD). WASI = Wechsler Abbreviated Scale of Intelligence, scores are presented as intelligence quotients (M = 100, SD = 15). The T-scores are age, gender and education corrected scores (Mean = 50, SD = 10). Abbreviations are as follows: IQ = Intelligence Quotient; NAART = North American Adult Reading Test; and WCST = Wisconsin Card Sorting Test.

The mean BDI scores were 13 ± 8.3 (range 0 to 39) and the mean BAI scores were 12.4 ± 10.3 (range 0 to 43). Twenty-six percent of patients had moderate to severe symptoms of depression and 19.6% had moderate to severe symptoms of anxiety. The PAH patients had decreased quality of life compared to normals as depicted in Table 3.

**Table 3:** Health-related Quality of Life Data

	**Total PAH group (N = 46)** ** Mean ± SD**	**Normals**	**Cognitive Sequelae (N = 27)** ** M ± SD**	**No Cognitive Sequelae (N = 19)** ** M± SD**	**Cognitive Sequelae vs. ** **No Cognitive Sequelae ** **P value**
**SF-36 Scores**					
General Health	37.6 ± 19.2	77	37.7 ± 18.6	37.6 ± 20.7	0.99
Physical Health	36.6 ± 23.5	82	35.7 ± 18.3	37.8 ± 30.3	0.78
Role Physical	37.2 ± 37.9	81	28.6 ± 35.2	50.0 ± 39.3	0.06
Role Emotional	69.6 ± 42.5	83	61.7 ± 46.0	81.5 ± 34.7	0.13
Social Functioning	55.1 ± 29.4	82	52.5 ± 25.6	58.7 ± 34.6	0.52
Bodily Pain	61.2 ± 24.3	79	60.8 ± 26.0	61.8 ± 22.3	0.89
Vitality	36.7 ± 22.1	65	35.7 ± 22.0	38.1 ± 23.0	0.74
Mental Health	52.0 ± 35.5	80	55.0 ± 33.9	47.6 ± 38.5	0.52

### Comparison of patients with and without cognitive sequelae

Patients with cognitive sequelae had significantly lower scores compared to those with no cognitive sequelae (Table 2) for: 1) verbal learning [RAVLT trial 1; F(1,44) = 4.45, p = 0.043 and RAVLT trial 5; F(1,44) = 6.98, p = 0.002]; 2) immediate verbal memory [LM I; F(1,44) = 7.68, p < 0.008]; 3) delayed verbal memory [LM II; F(1,44) = 9.91, p < 0.003]; 4) executive function [WCST perseverative errors; F(1,42) = 4.48, p = 0.04]; and 5) motor function [finger tapping right; F(1,41) = 3.78, p < 0.05, finger tapping left; F(1,41) = 5.22, p = 0.03, and grooved pegboard right, F(1,41) = 30.18, p < 0.001].

There were no significant differences between patients with and without cognitive sequelae for depression [BDI; F(1,43) = 0.003, p = 0.95)] or anxiety [BAI; F(1,43) = 1.04, p = 0.31]. There were no significant differences in SF-36 scores of the PAH patients with and without cognitive sequelae (p = 0.06 to 0.99; Table 3).

### Correlations

There were no significant correlations between any medical variable and neuropsychological test scores. There were no significant correlations between depression and neuropsychological test scores. Anxiety correlated with worse delayed verbal memory (RAVLT delay recall; r = -.36, p < 0.002) and working memory (Letter-Number Sequencing; r = -.35, p ≤ 0.02).

For the quality of life, worse working memory (Letter-Number Sequencing) correlated with lower Role Physical (r = .41, p ≤ 0.001). Anxiety correlated with lower Role Physical (r = -.41, p ≤ 0.001), Role Emotional (r = -.52, p ≤ 0.001), and Social Functioning (r = -.43, p = 0.005). Depression correlated with lower Vitality (r = -.44, p = 0.005) and Social Functioning (r = -.58, p ≤ 0.001).

## Discussion

Our patients' estimated premorbid IQ was in the high average range and was higher than their measured Full Scale IQ, suggesting a modest decline in intellectual function. Over half (58%) of our PAH patients had cognitive sequelae. The prevalence of cognitive sequelae in our PAH patients is similar to that observed following carbon monoxide poisoning [25]. obstructive sleep apnea [10], and following coronary artery bypass graft surgery [24]. The rate of cognitive sequelae was higher than one (45%) and two years (47%) after recovery from acute respiratory distress syndrome [24] but was similar to the rate (58%) found in patients with congestive heart failure [30,31]. Our PAH patients were assessed approximately 2.6 years post-PAH diagnosis and 70% had a NYHA functional classification of III or IV indicating they were in the advanced stages of PAH. It is possible that earlier in the course of PAH patients may have a less severe or a lower rate of cognitive sequelae. Possible mechanisms of the cognitive impairments include hypoxemia [32] and ischemia [30,31]. For example, patients with pulmonary disorders with concomitant hypoxemia have cognitive impairments, especially impaired memory and attention [33,34].

Our PAH patients have impairments in a variety of cognitive domains including 57% with impaired motor abilities, 40% impaired memory, 17% slow mental processing speed, 15% executive dysfunction, and 13% impaired attention. The PAH patients with cognitive sequelae neuropsychological test scores fell below the 8^th ^percentile of the normal distribution of cognitive function. The patients found activities that require memory, executive function, attention, and quick mental processing to be very difficult or impossible. For example, one patient reported that her memory problems caused her to make errors mixing her medication (e.g. flolan) as she would forget what step she had just completed. To prevent using incorrectly mixed medication, she would discard the medication and start over, resulting in wasted medication and increased medication costs.

No significant correlations between medical variables and neuropsychological test scores were found, likely due to a restricted range of scores as over half of the patients had cognitive impairments. One study found that quality of life was associated with etiology, NYHA class, chest pain, pre-syncope, abdominal discomfort, and distance walked in six minutes [35] however cognitive function was not assessed. It is unclear what role, if any, medical treatment may play regarding cognitive sequelae in PAH patients, as we did not assess cognitive function pre- and post-treatment. In addition we did not compare cognitive sequelae in patients on different medications, as our study was not designed to compare cognitive outcome by medication and we did not standardized treatment across patients. While PAH medications are associated with improvement in physical function and quality of life [36-38] the effects of treatment on cognitive function has not been studied.

Of our PAH patients 26% had moderate to severe symptoms of depression and 20% had moderate to severe symptoms of anxiety. Our findings of depression and anxiety are similar to previous reports in other pulmonary hypertension patients [36]. Depression secondary to medical illnesses is common and occurs in 10% of patients in primary care settings [39]. Depression has been reported in 25% of patients with chronic cardiac and pulmonary disorders [40] and anxiety in 10% to 40% of patients with pulmonary disorders [41]. Only a few studies have assessed the effects of treatment on depression or anxiety in patients with pulmonary hypertension. For example, patients treated with epoprostenol had a lower rate of depression and anxiety compared to patients who did not receive it [7].

Anxiety, but not depression correlated with worse verbal and working memory. There were no other significant correlations with any neuropsychological score. These findings suggest that anxiety, but not depression has a modest effect on cognitive performance, with verbal memory the most affected.

The quality of life in this cohort of PAH patients was lower than that observed in normal individuals [42]. The finding of decreased quality of life in our PAH patients is similar to that reported in other PAH patients [7]. Decreased quality of life has been associated with side effects of medications [43], diuretics [35], and higher NYHA functional classification [44]. Conversely improved quality of life has been reported following treatment with epoprostenol [7], sildenafil [45], inhaled iloprost [46], nifedipine [47], and lower NYHA class (classes I and II) [45].

Not surprisingly, patients with cognitive sequelae had lower neuropsychological test scores compared to patients without cognitive sequelae. There were no differences for depression or anxiety when comparing PAH patients with and without cognitive sequelae. Similarly there were no differences in quality of life in PAH patients with cognitive sequelae compared to patients without cognitive sequelae. Cognitive impairments are not associated with decreased quality of life in ARDS survivors [48] or medical ICU survivors [49]. In contrast, cognitive impairments are associated with decreased quality of life in ICU survivors with multiple trauma [50], in acute respiratory distress syndrome [51], following carbon monoxide poisoning [52], and in patients with chronic obstructive pulmonary disease [11]. Cognitive impairments are a major determinant in the ability to return to work, work productivity, and life satisfaction following acute respiratory distress syndrome [51]. Even mild cognitive impairments may result in problems in daily functioning such as driving, money management, and performing activities of daily living [53].

It is unclear why we found no relationship between cognitive sequelae and decreased quality of life in our PAH patients. One possible reason is that both the PAH patients with and without cognitive sequelae report very low quality of life resulting in a restricted range. Thus the low SF-36 scores occurred in all PAH patients regardless of presence or absence of cognitive sequelae resulting in non-significant differences. Another reason for the lack of relationship between cognitive sequelae and quality of life is that we did not use a quality of life instrument specific to PAH. A quality of life instrument specific to PAH might better assess and detect relationships between quality of life and cognitive sequelae.

The strengths of this study include a prospective cohort study design; assessment of oxymetric oxygen saturation and heart rate pre-and post-neuropsychological testing, comprehensive neuropsychological measures administered in person, and a well defined PAH population.

Limitations of this study include the lack of an appropriate control group from which to make comparisons. However, we used demographically corrected normal population data for (i.e. age, gender, and education) to control for factors known to affect cognitive performance resulting in improved neurodiagnostic accuracy. A second limitation is the inability to measure premorbid cognitive and emotional function, and quality of life similar to other studies in acute and critically ill patients. A third limitation is that the observed cognitive sequelae may have been due to factors other than PAH including age, premorbid cognitive impairments or comorbid medical disorders. Evidence suggesting that the cognitive sequelae are not due to the above factors include 1) It is unlikely that the observed cognitive sequelae in our PAH patients were due to pre-existing cognitive impairments because we screened for patients with prior cognitive impairments. 2) Our patients are younger (mean age 48 ± 12 years) than typically associated with age-related cognitive impairment or dementia (age ≥ 65 years) and there was no difference in age of patients with and without cognitive sequelae. While it is possible to use surrogate screening tools to detect premorbid cognitive impairments, these instruments are designed for use in older patients (age ≥ 65 years) which is significantly older than most of our patients. 3) Our patients' education level was high (13.6 years) and there was no difference in education level in patients with or without cognitive sequelae, thus low education level cannot account for the observed cognitive impairments. 4) Few patients had comorbid disorders associated with cognitive impairments. Nine patients had obstructive sleep apnea identified at PAH diagnosis, all of whom were undergoing treatment at the time of neurocognitive testing, making it difficult if not impossible to separate the cognitive effects of PAH and from those of obstructive sleep apnea. Two patients had diabetes and two had systemic hypertension, all which were treated. Even if all four of the patients with diabetes and systemic hypertension had cognitive sequelae, it would not account for the high rate of cognitive sequelae in our PAH patients. Therefore it is unlikely pre-existing cognitive impairments account for the observed cognitive sequelae in our PAH patients.

## Conclusion

Pulmonary arterial hypertension patients have cognitive impairments, depression, anxiety, and decreased quality of life. We found no differences for depression, anxiety, or decreased quality of life for patients with cognitive sequelae compared to those without cognitive sequelae. Decreased quality of life was associated with worse verbal and working memory. Clinicians should be aware of adverse brain related outcomes in PAH patients. Attention to proximal determinants and possible interventions to prevent or reduce cognitive and emotional morbidity and decreased quality of life are warranted and should be an emphasis in outcomes research.

## Competing interests

The author(s) declare they have no competing interests.

## Authors' contributions

JW, ROH, and CGE designed and ROH coordinated this study. JW and ROH performed and interpreted the neuropsychological, depression, anxiety and quality of life assessment. EWG performed data entry, data checking, and database management. JW and ROH carried out and interpreted the statistical analyses. CGE and NK recruited patients, obtained medical data and assessed results. The manuscript was written by JW, ROH, and CGE. All authors read and approve the final manuscript.
